# Re-Organization of the Illinois State Dental Society and the Application of the Plan in Michigan

**Published:** 1907-01-15

**Authors:** Arthur D. Black


### RE-ORGANIZATION OF THE ILLINOIS STATE DENTAL SOCIETY AND THE APPLICATION OF THE PLAN IN MICHIGAN.

ARTHUR D. BLACK, B.S., M.D., D.D.S.

Read before the Michigan State Dental Association, July 9, 1906.

If we look about us in this busy world to-day and compare it with what it was a dozen or more years ago, we find that radical changes have been made in the methods of doing things. Almost every branch of business has been re-organized and systematized to keep pace with wonderful growth and development. Our educational systems have been greatly modified to enable larger numbers to enjoy the benefits of higher education and systems have been introduced which give opportunity for selective study for particular fields of work. The annihilation of space by modern inventions has brought us more closely to each other and readjustments have been necessary everywhere. The professional man must keep up in the race for progress. There has been a gradually increasing demand for better methods of advancement by professional men; more interest is being taken in society work; there is a desire in the hearts of most men to keep up to date and they are looking more and more to societies as the means of doing so. As a result of this desire the medical profession has carried on a wonderful campaign of organization which has without doubt resulted in more prompt and wider dissemination of new thought and correspondingly better service to the public. State after state has been organized, all on the same general plan, under the guiding hand of the great central body, the American Medical Association. They have applied business methods to society work and the recruits have come in by hundreds and thousands.

The feeling among the members of the dental profession is not different from that of the medical. There is a decided increase in interest and in the number of members in dental societies all over the country. The men who are not members need only to be approached in the proper way and they gladly join. Unfortunately we have no national body which seems to be capable of taking up this work as has the American Medical Association. As many of our state organizations are more efficient than our national body it is natural that this work should be undertaken by our most progressive states independantly. The air was so full of the work of the Medical Association in Illinois that we followed their example and re-organized our dental societies two years ago. We found the profession of our state so ready to accept the plan that we were able to organize the entire state within a single year, increasing our membership from less than four-hundred to over thirteen-hundred, and the past year has added more than another hundred.

We have received inquiries regarding this plan of organization from many states and believe that the next few years will bring about a much wider interest in society work. In its most essential points, the plan followed in Illinois should be applicable in other states and it is a pleasure to give the members of the Michigan Society a brief review of what we have done, with a comparison of conditions in our state before and after the re-organization.

As to the plan itself, it is hardly desirable to give more than a general idea of it in this paper. I have brought copies of the reports of our re-organization committee with a number of printed blanks, which give in detail the work that has been done, together with its cost.

These will be handed to your Secretary.

In 1904 there were about three thousand dentists in Illinois and we had a membership of less than four hundred in our State Society. There were a number of local and district societies scattered throughout the state, none of which was in any way connected with the state organization. The total membership of these local societies was considerable, yet there was no possibility of united action by them. The first work of our re-organization committee was to make up as accurate a list as possible of the dentists of the state, and mark all who were members of any dental organization. These lists were arranged by counties and were sent out to be checked over by selected men, who were requested to mark all who would be desirable as members. After this had been done, we were ready to begin the work of organization. The state was divided into thirty sections, varying in size from one to ten counties, according to the number of dentists and the transportation facilities. Committees were appointed in each of these sections and meetings for organization were held. A form of constitution had been previously prepared, so those adopted by all local societies were practically the same. Nothing was left undone to make these meetings interesting, men from distant parts of the state were sent to address those present and assist in the details connected with organizing, and in many cases a majority of the practitioners of a section were present.

The relation between the local and the State Society is the most important feature of the plan. The local society is the portal of entrance to the State Society in its territory: it passes on all applications for membership and may expel men residing in the territory over which it has jurisdiction.

On the other hand each member of every local society is necessarily a member of the State Society; the dues paid to the local secretary cover both, so a man cannot belong to one and not the other. This gives a large central body composed of definite units, each of which cares for its own welfare and that of the organization in its territory. Aside from regular society work much may be accomplished by such an organization. Let me mention one instance; when our new dental law was apparently hopelessly pigeon-holed by a committee to which it had been referred and the efforts of our political friends had failed to get it reported out, the officers of each local society were requested by telegraph to get their members to write and telegraph their representatives and in less than forty-eight hours about a thousand letters and telegrams were sent in and our bill was promptly reported out.

There is a difference between the small State Society composed largely of the most progressive men, and the large society, composed of half of the profession of the State, which should not be overlooked.

Up to the last few years, most of our state dental societies were composed of about ten percent of the practitioners, who were generally the men of greatest activity in all lines: who were doing the most advanced work. These men undoubtedly were benefited by the society membership and their work had a certain influence for good in the general advancement of matters of interest to the profession, but how much were the other ninety percent getting from the society? Very little of actual practical value. In the larger organization composed of fifty per cent of the practitioners there will naturally be a wider distribution of thought and ideas, but the deveolpment of advanced scientific research may be temporarily retarded somewhat in exchange for the general advancement of a larger number of men. We are of the opinion in Illinois that within a few years there will be developed in our state a much larger number of strong men, and a material improvement in the service rendered by the average practitioner will naturally result. We believe that our State Society will be of the greatest benefit to the people of our state under the present organization. We are developing a very close relationship with the Illinois State Medical Society, the Pharmacists, the Nurses and similar associations, through which any one of these will have the support of all measures requiring such support and can muster some ten thousand members of these organizations.

The ultimate success of a State Society so organized depends very largely upon the local societies of which it is composed. The local society should be of the greatest value in the development of men; the members should get more from the local than from the Sate Association. The members of each of these components are facing about the same problems, the same business conditions. These men meet occasionally and discuss both scientific and business topics at close range; there is a better understanding among them.

The natural result of these meetings is the establishment of a feeling of good fellowship which is probably more important than all else, and will do more to elevate dentistry.

The more considerate each man is in speaking to a patient of his brother practitioner, the higher opinion that patient will have of the dental profession. Another feature of our local society work has been the frequent exchange of visits by the men of the various sections and our State Society pays the expences of one man, selected by the local society, to read a paper before them each year. We are in in this way developing a closer acquaintance of the men throughout our state and the feeling of good-fellowship is broadening.

I wish to make a few suggestions which may be of value, should the Michigan Society decide to take up this work of organization. Make good-fellowship and harmony the basis of your work. I believe failure or success depends primarily on this. If there is friction anywhere it must be eliminated.

Every man should be willing to sacrifice some personal desire or feeling for the advancement of the cause, and those who cannot prosecute their part of the work without friction should refrain from taking an active part. If an organization is built up along the lines of harmony and good-fellowship, all other matters will be easily taken care of as a natural result.

Let me warn you that the work of organization and the conduct of such a large society is quite a large proposition, and requires systematic business management. The active men of the state organization must keep in close and constant touch with the components. They must know what each is doing. If there is a lack of interest anywhere, action must be taken to stir things up. If a local officer is not doing his duty he must be impressed with the importance of his position and the necessity for action. Local membership committees must be constantly urged to look for recruits and the local secretaries must urge promptness in the payment of dues. In Illinois this year the State Society had in its treasury the dues of about twelve-hundred members a month before the time of our annual meeting. We do not count a man a member any year until his dues are paid. We publish a little official monthly bulletin which enables the officers and members of the various local societies to know what the others are doing. This stimulates a friendly rivalry and materially assists in keeping up interest.

In closing let me call your attention to the possibilities of our having a large and truly representative national organization in the near future in case a considerable number of states adopt this plan We should have a national association with from fifteen to twenty-thousand members instead of a weak organization of a few hundred.

Many states have either begun work or are considering the matter and from the letters we have received, we are led to believe that the next few years will bring about a wonderful change in the dental organizations of this country.

It was understood between Dr. Essig and myself that Dr. Essig would present the part of this subject that referred particularly to conditions in Michigan and simply to the application of this plan in Michigan, so that I have not had much to say in this paper in reference to conditions in this state but it is rather more of a general statement as to the plan that we have followed in Illinois and the results that we have attained there. I have purposely made this paper quite short for the reason that I hope there is enough interest in this matter in this state so that there will be considerable discussion of it, and I will be pleased if I may in the closing discussion have something to say in reference to conditions in Michigan as I have learned them since I arrived here this morning.

DISCUSSION.

Dr. C. H. Worboys, Albion: W expected a good deal from the meeting of the re-organization Commitee and we should have had a good report to make, had the paper that Dr. Essig has prepared, come to hand. I can only say that so far as I know we would recommend following the Illinois plan.

The State of Illinois is divided as to the congressional districts, while Michigan should be according to population.

I think the Illinois Division is a little better. Dr. Essig thought it better to make our districts on the basis of practicable accessibility.

The congressional districts may be alright from population, but means of transportation is much more important in our state. Through the southern part of our state it makes very little difference how you divide it, because we can travel on steam, trolley, or good wagon roads; but, when you come to the northern part of this state conditions are entirely different. I think, however, all these things will prove satisfactory, should we decide to undertake to carry out this idea I wish to call your attention to the fact that we have representatives of some half a dozen different cities of the state, and I believe it would be to our interest and the interest of the whole profession to get a word from some of those representatives of the different societies. This thing has been talked over among them, and let us hear how they feel about it and what they will do. It will be something of a starter if a half dozen of the existing societies take up the work willingly and are willing to back it up and break up some of their organizations, which will be necessary.

Dr. N. S. Hoff: I think re-organization would be one of the grandest and best things we could do for the profession in this state. As we are scattered over this great state some dentists in the upper peninsula are so remotely situated that they cannot easily get to this Convention held in Detroit.

It seems to me that this plan of having local and district sociteies is a practical and important movement for us to undertake. Illinois is not a difficult state to get over from any part of it but there are situations in this state that are absolutely unreached in a professional way by professional societies, and I do not believe that there is anything this Society can do that will be of such benefit to all the members of our profession as this plan of re-organizing our work and making it more effective.

I liked the statement of Dr. Black in regards to the matter of organizing these societies and having them visited periodically by men from other sections of the state. It struck me as a very happy idea, and one that had in it the elements of great success in developing the local Society idea. It is kind of a habit, this idea of going to society meetings, and some of us have the habit and others do not have it. Lots of men in Detroit are not here tonight because they have not this habit. It is a habit that we ought to cultivate for the good that we can do each other and the people.

We can all get together in these local societies and discuss the principles that we are working with day by day with much benefit and each one will get some benefit, we shall get the most benefit if we participate in the discussions. A discussion of the most trivial thing will help us intellectually and stimulate us to think. The more benefit we ourselves receive from such intercourse the more able we shall be to render that service to the public which the public wants, and sadly needs, and which we are not rendering. I believe it is high time that we were waking up to the fact that as a profession we do not know it all, and the more we discuss our work together, the more we shall help each other.

I do not believe in combining ten counties into a section. It may be that there may be sections in the North where it would be necessary; but if we could get not more than three or four men together in a dental society they could have a good session, and they would be better men for it.

I think we ought to organize more societies and localize them, so that they could have opportunities to cultivate their profession and get better acquainted and become harmonious in their business methods if nothing more. This would tend to elevate the dignity of our profession, as well as the worth of our calling, so I am heartily in favor of anything that will help us to organize. In regard to the kind of a man that should be sent around to visit these societies. He should not be some great star who will set them on fire, some man from abroad, but a man from our own state, who is a member of this society. We should get our own men to going about doing this work, for by putting upon them the responsibility of trying to uplift somebody else, this will help to develop them faster than anything else. The idea of having someone come from outside is a very valuable one, used occasionally, but it is not so good as it is to have our neighbor from across the street come into our office and sit down and talk to us and tell us how he does it, and what success he has; which is not the same thing as coming to this meeting or or even going to a little local society where we are not afraid to talk as we would be if there were a great star present talking to us, we simply sit in our chairs and do not have the courage to get up and criticize or even question him. By such a plan we shall develop a great interest in those places where the practice has now practically nothing to stimulate to better effort. We all want to get into condition where we feel that we are on earth for a purpose, and our purpose is one of the noblest of all purposes, that of serving mankind in the best possible way. I do not think we can get this idea into our profession too soon or too thoroughly. I think this idea of having the local societies working under the auspices of the state society, making a complete organization, will help to develop greater skill and ethical professional customs and relations.

DR. BLACKMARR, Jackson: I am a member of the local Society of Jackson, and we have taken this subject up with a great deal of enthusiasm.

Dr. Howlett has been greatly interested in learning the workings of the medical societies along the same line. We are very much enthused over this. It is one of the grandest movements that we have had. I was disappointed to night to think that we could not hear Dr. Essig. From talking with other members of our Society, I never have heard any dissensions in regard to this, nothing but praise.

Dr. Morse, of Lansing: Those members of the Central Society to whom I have talked in regard to the matter seem to have been much pleased with it, and we think it would be an excellent idea to adopt the plan as suggested.

Dr. C. J. Gray: I do not think there is a local dental society north of a line drawn from Saginaw to Grand Rapids, nor in the Upper Peninsula. I think the possibilities would be good for organizing some but the conditions are different from what they might be in a large part of the state as in Central or Southwestern Michigan. In that region, a large part of it at least as on the shores of Lake Superior and around from Alpena, and along other shores they are given up to summer resort business, and consequently in July, August and September it would be absolutely impossible to hold dental society meetings outside of your local town. In February and March you cannot probably get enough together with any degree of certainty to hold a respectable meeting; so that for, about six months or half of the year, you are practically knocked out. There was a Northern Michigan Dental Society organized about two years ago, but they started in the month of July, organized by Cadillac and Traverse City parties; it was intended to take in a district comprising Big Rapids and as far as the Straits, including all the upper lines of railroad. I believe there were only six or seven there when the Society was organized Since then some of the members have moved away and nothing has been done. I believe that an organizing force which could be appointed to organize the state would meet with good success, if taken at the right time. We are in the back woods up there, but I know whenever I come down to these state society meetnigs I never see more than a couple of upper peninsula men here.

Dr. Worboys: If we were to organize according to the Congressional Districts, and according to the population, that part of the work is already done. I cannot see why we cannot adopt Dr. Blacks methods, because nearly all the preliminary work is done except to appoint our workers in the various districts. We cannot tell who to appoint until we get Dr. Essigs report, which gives the town and the number of dentists in each and also the names of the men in these districts, who will work. Then the appointment of these men in the several districts will insure the completion of the work. I think if we make up our minds to do this thing we can. If we want to take up this matter this is the time to do it, not next year. I know the districts that I am in will furnish plenty of capable workers, and in other districts there are plenty of good workers.

The time and place of meetings for the various local organizations can be decided on the basis of whatever is most suitable for each particular district. They need not all have meetings at the same time. Local organizations need not disband or give up their society; this plan need not effect their present methods. This aspect of the case, interests me very much, because I know in the Southwestern Michigan Society the members are rather proud of their society, they are a lot of willing workers and I do not think they want to give up, but they will work in districts for the State Society. If the State Socity is formed of district societies it will be only a matter of a few years before there will be no other organization in the state. We need no other, so I think that now is the time to get at this.

Dr. Hoff: It seems to me that the great difficulty in adopting this plan of organization is that we shall have to get the consent of the other societies. As I understand it, the State Society is supreme in all matters. It dictates to a certain extent what each local society shall do, not exactly in the way of what they shall say or do with their programmes from time to time, but it at least compels them to contribute

$2.00 per year to the State Society. Whether they will be willing to do this or not is the problem in my mind.

Dr. Black: What is the function and duty of a State Society anyhow? Is it not to do everything that will better the dental service to the public in the particular state? It is not a question of whether fifty or one-hundred men get together in an organization, it ought to do that which will improve the dental service of the state, and if you can make one-half or three-fourths of the dentists of the state better, you will raise the average of the whole state and the public will get better dental service in that way than to have 150 or 200 star performers and the rest scrubs. So there can be no question but what the plan that will bring most men into adental organization will be the best thing for the people.

The conditions in Michigan, as I see them, are not particularly different from what they were in Illinois. I believe if anything we had more troubles than you would have here.

You have in this state a State Dental Association which meets once a year, a Central Michigan Association which meets once a year, and the Southwestern Michigan which meets once a year. These might be considered as District Societies. Besides that you have a number of District Societies, the first district and the Southern district society in addition to a number of city societies.

In the first place, take the city societies. We asked each city society to adopt the constitution which was prepared and there was nothing in it to which there was any objection. We asked each city society in adopting this to change from a city to a county organization, that is, to take in just a little more territory than they had before. We had absolutely no difficulty with any of the existing city societies in their doing this. That takes care of the city organizations. In the district societies we had a great fight with our Northern Illinois Society. One of our old members came and said it would kill the district society. He put up a terrible fight.

We tried to show him then that it would be a good thing for the Northern Illinois district society, and as a matter of fact we did not ask that society to take any action whatever. We went out during the year and got every man with the exception of this one into the various local societies in that district. In addition to that we got some 200 more who were not members of that district society, so that instead of 140 society men in that territory at the end of the next year we had 340 men who were society members. The natural result was that when that district society met next year they had a larger membership than ever before, although still independent of our organization. Your societies can retain their organization just as they are now, either independently or in connection with it. The district societies may become a part of this state organization whenever they agree that all of their members must be a member of the state organization.

Supposing in the territory of the Southwestern Michigan there are three or four local societies and if they include all the members of the district society they will be all members of the state society. They can get together and have their district meeting just as before. Now each district society will be a combination of the four or five local societies that happen to be in that territory. The district society retains its identity just as it did before. The Southern Illinois Society meets this year in the fall and for that meeting a number of the local societies occupying that territory simply skip one of their local meetings and they all meet together as a district society and it has not interfered in any way with any society that was then in existence.

The business of our State Society is conducted entirely by a council of 12 men, three of whom are elected each year.

These twelve men transact all the business of our state Society. None of it is done by the general body. From this committee of twelve they appointed a committee of three to supervise this re-organization work and represent the council. The work itself was done by a re-organization committee of three men and this re-organization committee was subordinate to this general committee of the council.

The Society as a whole paid no further attention to it, except that the members of each society work in harmony with our committee.

In regard to the meetings of these local societies. That is left absolutely to each local society, and it is a question of the conditions existing in the district. Take it in the Northern peninsula of Michigan, I should say that it would be very desirable to include a larger territory into each society and have their meetings less often. In the Southern part of Illinois we have many counties which will not average more than three or four members in the county. You ought to have 15 or 20 men in each society to make it a good one. If the men live so far apart that they cannot get together frequently, then make the meetings less frequent. Most of our mistakes in organizing were in having these meetings too frequently. Some wanted them once a month. They did that for a few months and then changed to four times a year. In those sparsely settled portions of the state, two or three of the societies have only a meeting in the fall, the intention being that they will attend the state society meeting in May. One meeting of the local and one of the State Society. That is a thing that ought to be settled by each local society, and just as suggested by your committee, I would say that these locals when organized ought to be urged not to have their meetings too frequently.

We have within one of our sections in Illinois a section which includes two or three counties in which there are three large towns in the section and they did not want it divided up into three sections, so we made one section of it which meets twice a year and in those cities they organized what they call "Fellowship Clubs" where the members get together about once in six weeks. You can not possibly organize any whole state on exactly the same plan for each section.

Change your plans in each section to fit the conditions there.

There are three things that I think ought to be done in connection with this work to keep it going and keep it up in good shape.

First, there should be a committee to keep in close touch with those various local organizations, and I believe you should publish some kind of a bulletin from the organization committee, which will go to all the members every month or every quarter containing notices, announcements of meetings and brief reports of the meetings that have been held. That is a cheaper method of getting at your members than circular letters: you can send it as second class matter and the postage is practically eliminated. It keeps up the interest, and not only that, it keeps poking everybody up. For instance, if you are publishing this monthly bulletin the secretary or whoever publishes it writes to the local society secretary asking him to send in his program for the month or year. This in many instances the local secretary will have neglected to prepare, and it will be a means of poking him up, in a nice way, and he will get at it and send it in. Or, if some journal could be used in the same way with a few pages in the back as a supplement to take the place of the bulletin, it would be just as well.

Another thing would be to get some one to visit the section meetings (in certain sections where it is needed) who understand the detail of the work and can help in the organization, and explain what they ought to do.

The State Society gets $2.00 dues from each member, and each local society can add as much to that as maybe necessary for their own support. In most local societies they charge $3.00. The secretary keeps one dollar and sends $2.00 to the State Society. Some charge $4.00 and some $5.00. For the $3.00 they get membership in both the local and the state Society. In addition to that, we have a contract with the Dental Review by which that magazine is sent to each member of the society every month in the year at a cost of 50 cents per year. This journal publishes all the proceedings of the State Society and Chicago

Dental Society and eight papers that are sent in by our Committee. We feel that we are giving the members a good deal for the money, perhaps a little too much.

To my mind, the greatest thing of all we have accomplished in the State of Illinois has not been anything we can show you by figures but in the difference of feeling that exists in the profession of that state. In that connection an incident comes to my mind Back in Rockford a lady had a crown put on, which she did not like. She went to another dentist and talked about the man who put the crown on, stating that it was not satisfactory, but got little sympathy. She went to another dentist in like manner but they failed to sympathize with her complaints, but advised her to go back to the first dentist and have him make the crown right. She finally saw that she would have to go back to Rockford. They treat each other right, and they have that feeling which is broadening all over our state, the feeling of one dentist for another, a fraternal feeling which is entirely different than it was before.

DR. PROTHERO. I have been called on to visit a number of the local societies, and among others quite a number of Chicago men have been asked out, and in visiting the local societies we have noticed that where there has formerly been a great deal of ill feeling among the members of the profession, they have now gotten together and it is now more like a fraternal society than anything else. It has done a wonderful lot of good in our state. The men are better dentists and give better service to the communities in which they live: and they are respected by the public. It is a work worthy of every effort on your part to re-organize your society and get everybody in line, do it now.

DR. BLACK: The question of financing the work has to be considered and also the question of getting the members. You cannot possibly tell or know how successful this work will be, and it is not possible to make any kind of an appropiration now or estimate as to what might be done, but it is certainly not necessary for you to organize the entire state in one year. When we first started out we planned to take three or four years to organize our state. We picked out the easy fourth first and then spread out a little more and more until finally we saw things were coming in our way so nicely that we did it all up in one year. We started out with the intention of spending $600.00 of the Society's money. The dues of the members came in so rapidly that you can easily see we increased our dues to $1300 or $1400 the first year, and if we had only spent that money the first year the Society would have been nothing out.

I would suggest that this Society should appoint an organization committee of three to five men, and that you should appoint three other men, possibly your older men, to whom this committee will be subordinate on the financial proposition. It is too much to ask of a committee to do all of this work and make them responsible entirely for the finances. Have this other committee with whom they may consult on any movement which will involve more expenditures. Let that committee of three in a way represent your whole society and act for the society during the interval, so that the organization committee, when any proposition comes up, can advise with it I think if we had not had that other committee back of us we would have hesitated about going ahead on some important propositions. You can take any amount of money that is available and use it up, and see what the results will be, and then plan to go on further.

Dr. Hoff: I move you that it is the sense of this meeting that we should re-organize the profession of this state in accordance with the plan of the Illinois Dental Society, and that the Board of Directors be authorized to raise a committee of three to supervise the work for this re-organization plan during the coming year. Motion Carried.

